# Macrophage metabolic reprogramming in sepsis-associated acute lung injury: mechanisms and therapeutic strategies

**DOI:** 10.3389/fimmu.2026.1808474

**Published:** 2026-05-15

**Authors:** Ran Pan, Yiyi Sun, Lu Chen, Jianping Pan, Junping Guo

**Affiliations:** 1 Rehabilitation and Nursing School, Hangzhou Polytechnic University, Hangzhou, Zhejiang, China; 2 School of Medicine, Ningbo University, Ningbo, Zhejiang, China; 3 Department of Clinical Medicine, School of Medicine, Hangzhou City University, Hangzhou, Zhejiang, China

**Keywords:** Acute lung injury, macrophage, metabolic reprogramming, sepsis, therapeutic targets

## Abstract

Sepsis-associated acute lung injury (S-ALI) remains a life-threatening condition with high mortality and limited therapeutic options. Macrophages, as key sentinels of innate immunity, exhibit remarkable heterogeneity and functional plasticity. These properties are fundamentally driven by metabolic reprogramming, which tailors their effector functions to specific microenvironmental demands. Beyond the traditional M1/M2 binary classification, macrophage activation is now appreciated as a continuous functional spectrum. Pro-inflammatory macrophages preferentially utilize aerobic glycolysis and the pentose phosphate pathway, coupled with suppressed oxidative phosphorylation (OXPHOS), whereas reparative macrophages rely predominantly on OXPHOS and fatty acid oxidation (FAO). Key glycolytic enzymes such as PFKFB3 and PKM2, the transcriptional regulator HIF-1α, and TCA cycle intermediates including succinate and itaconate serve as critical metabolic checkpoints governing macrophage inflammatory responses. During S-ALI, the metabolic landscape undergoes dynamic temporal shifts: the early hyperinflammatory phase is characterized by enhanced glycolysis, while the late immunosuppressive phase exhibits impaired OXPHOS and FAO. This review synthesizes recent advances in understanding how metabolic reprogramming orchestrates macrophage polarization during S-ALI, encompassing glycolysis, the TCA cycle, FAO, and amino acid metabolism. Natural compounds, pharmacological inhibitors, and innovative delivery platforms have shown promise in reprogramming macrophage metabolism to restore immune homeostasis. Notable examples include aerosolized CRISPR/Cas9 nanotherapeutics, biomimetic nanoplatforms, pH-responsive nanoparticles, and engineered exosomes. However, challenges such as broad cytotoxicity, limited macrophage selectivity, incomplete pharmacokinetic characterization, and the timing of intervention in the evolving septic milieu must be addressed. Future strategies should focus on developing cell-type-restricted delivery systems, validating targets in human-relevant models, and designing phase-specific interventions tailored to the metabolic trajectory of S-ALI.

## 1 Overview of sepsis-associated acute lung injury and the immunometabolic role of macrophages

Acute lung injury (ALI), a frequent and severe complication of sepsis, is a major contributor to short-term mortality and long-term impairment in survivors (1). A wide range of intra- and extra-pulmonary insults, including trauma, ischemia-reperfusion injury, shock, pneumonia, drug toxicity, and sepsis, can precipitate this hypoxic respiratory syndrome (2–4). These diverse pathogenic factors collectively disrupt the integrity of the capillary endothelial and alveolar epithelial barriers (5), and, if unresolved, can progress to acute respiratory distress syndrome (ARDS). Pathological features of ALI are characterized by excessive lung inflammation, endothelial and epithelial damage, increased vascular permeability, and diffuse alveolar injury (4, 6–10). Clinically, patients present with diffuse bilateral pulmonary infiltrates, profound hypoxemia, and pulmonary edema (11). Epidemiological data show that approximately 68.2% of patients with sepsis develop ALI, with a 90-day mortality rate of 35.5% among those affected (12). Despite extensive research, no effective therapies are currently available to reduce mortality or improve the long-term prognosis of ALI patients (13).

**Figure 1:**
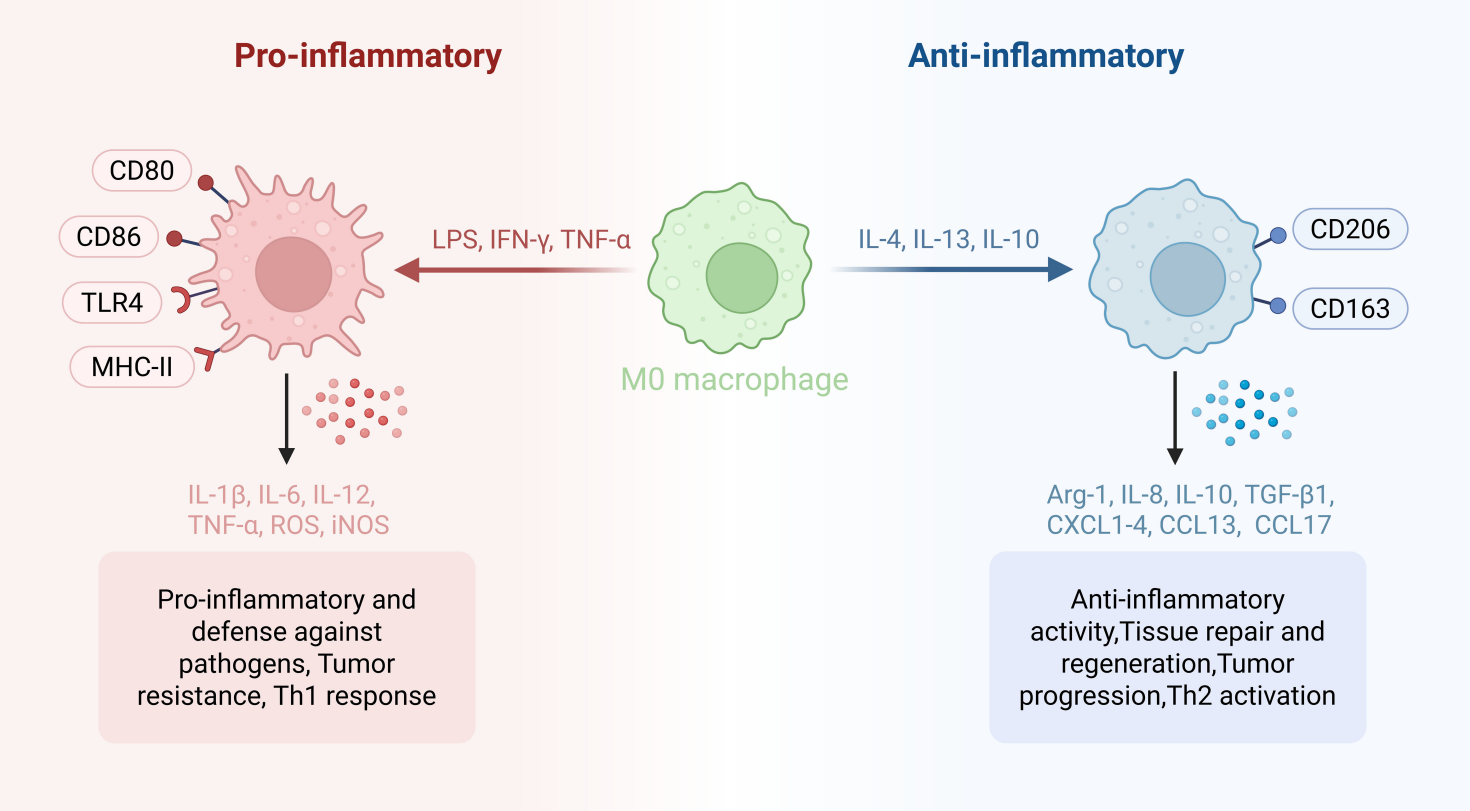
The phenotypic spectrum of macrophage polarization. Macrophages exhibit remarkable functional plasticity and can adopt a continuum of polarization states in response to diverse microenvironmental cues. At the extremes of this spectrum, macrophages activated by Th1-associated stimuli such as LPS and IFN-γ acquire pro-inflammatory effector functions characterized by the expression of surface markers including CD80, CD86, and TLR4, and the secretion of inflammatory mediators and cytokines. These cells are essential for host defense against pathogens and tumor resistance. Conversely, macrophages exposed to Th2-associated cytokines such as IL-4 and IL-13 adopt reparative and immunoregulatory functions, expressing markers such as CD206 and CD163, and producing anti-inflammatory mediators and cytokines, which promote tissue repair and inflammation resolution. It is important to note that this binary representation is a simplified framework; accumulating evidence indicates that macrophage polarization exists along a dynamic continuum, with intermediate or hybrid phenotypes shaped by the integration of complex signaling and metabolic cues. Created with BioRender.com.

Macrophages, as essential sentinels of innate immunity, are distinguished by their remarkable heterogeneity and adaptive plasticity, which enable them to coordinate tissue homeostasis, orchestrate inflammatory responses, and facilitate repair processes across diverse organ systems (14, 15). Macrophage populations arise from two principal developmental trajectories. Tissue-resident macrophages (TRMs) are seeded during embryogenesis and sustain themselves locally through *in situ* self-renewal, whereas monocyte-derived macrophages (MDMs) originate postnatally from bone marrow precursors and are actively mobilized to inflammatory or injured niches via the circulation (16–19). Together, TRMs and MDMs constitute an integrated and highly adaptable cellular network that underpins both immune surveillance and tissue restoration (14, 16). In the context of activation, macrophages were traditionally classified into two polarized states: classically activated pro-inflammatory M1 and alternatively activated anti-inflammatory/reparative M2 macrophages, which represent functional extremes of a phenotypic spectrum (20, 21) (**Figure 1**). M1-like macrophages, typically induced by IFN-γ or LPS, display strong antigen-presenting capacity and secrete pro-inflammatory mediators including IL-1β, TNF-α, IL-6, IL-12, iNOS, and ROS, thereby promoting Th1-type immunity and pathogen clearance (21–29). In contrast, M2-like macrophages arise in response to Th2-associated cytokines such as IL-4 and IL-13, express markers including Arg-1 and CD206, and contribute to tissue repair, angiogenesis, and inflammation resolution (19, 30–33). Yet, accumulating evidence from high-resolution transcriptomic analyses has rendered the strict M1/M2 dichotomy obsolete. It is now appreciated that macrophage polarization encompasses a fluid continuum of functional states, dynamically sculpted by the convergence of contextual signals, intracellular signaling cascades, and chromatin-level regulation (34–36). In sepsis-associated ALI (S-ALI), this complexity is further amplified by the dynamic recruitment and polarization of distinct macrophage subsets within the injured lung microenvironment.

The functional plasticity of macrophages is fundamentally driven by metabolic reprogramming, adaptive changes in cellular energy metabolism that support their diverse effector functions (37, 38). Key metabolic pathways involved include glycolysis, the tricarboxylic acid (TCA) cycle, the pentose phosphate pathway (PPP), and fatty acid and amino acid metabolism. Pro-inflammatory macrophages preferentially utilize aerobic glycolysis and the PPP, coupled with suppressed oxidative phosphorylation (OXPHOS), to fuel the rapid production of inflammatory mediators and ROS (23, 37, 39, 40). In contrast, reparative macrophages rely predominantly on OXPHOS and fatty acid oxidation (FAO) to sustain their tissue repair and immunoregulatory functions (41, 42). Specific metabolic intermediates also can act as regulatory checkpoints that modulate macrophage polarization and effector functions through diverse signaling mechanisms (43, 44). An imbalance in M1/M2 macrophage polarization is closely associated with numerous inflammatory conditions, including S-ALI (45). During the early stage of S-ALI, the lung microenvironment becomes dominated by pro-inflammatory macrophages and their mediators, driving hyperinflammation, endothelial injury, and organ dysfunction (29, 45–47). Moreover, endothelial-derived extracellular vesicles further aggravate S-ALI by reprogramming circulating monocytes toward a pro-inflammatory phenotype (48). As sepsis progresses, a shift toward an immunosuppressive phenotype occurs, characterized by impaired OXPHOS and FAO, reduced anti-inflammatory cytokine production, and delayed inflammation resolution, features that contribute to the late immunosuppressive phase of sepsis (23, 41, 42, 49–51). The controlled transition between these functional states is therefore essential for restoring immune balance and promoting tissue regeneration in S-ALI (19, 49, 52). Therapeutic strategies aimed at modulating macrophage metabolism, including mesenchymal stem cell-based approaches and targeting of metabolic checkpoints, have shown promise in shifting the balance toward reparative phenotypes and improving outcomes in preclinical models (53–56). In this review, we summarize current understanding of how metabolic reprogramming shapes macrophage behavior in ALI and discuss emerging therapeutic strategies aimed at harnessing macrophage immunometabolism for the treatment of S-ALI.

## 2 Glucose metabolism

**Figure 2:**
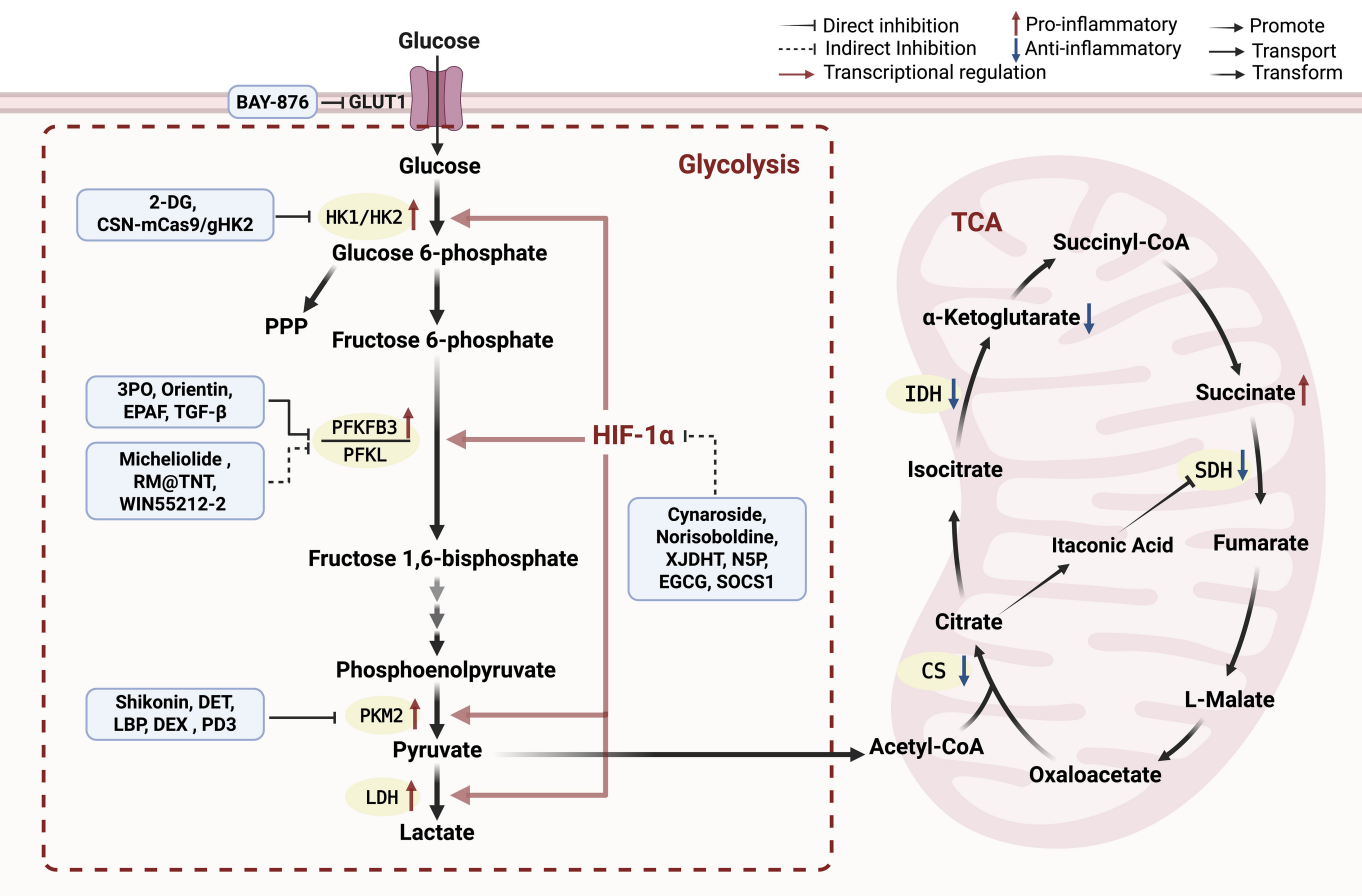
Reprogramming of glucose metabolism during macrophage polarization. Glycolysis is upregulated via glycolytic genes (HK1, LDH), PFKFB3 and PKM2, which enhance flux through the pathway and promote macrophage pro-inflammatory polarization. Natural compounds or traditional Chinese medicine ingredients such as orientin, micheliolide and shikonin directly or indirectly modulate metabolic enzymes including PFKFB3 and PKM2, while compounds such as cynaroside and XJDHT regulate HIF-1α-related pathways, both of which suppress glycolysis and inflammatory activation. Emerging delivery technologies, including CRISPR/Cas9 nanoplatforms, biomimetic nanoplatforms, and pH-responsive nanoparticles, are opening new possibilities for achieving macrophage-selective metabolic targeting. During macrophage pro-inflammatory polarization, the TCA cycle displays characteristic interruptions at isocitrate dehydrogenase (IDH) and succinate dehydrogenase (SDH), leading to the accumulation of citrate and succinate; the accumulated citrate subsequently fuels itaconate production. Targeted modulation of IDH and SDH may represent a novel therapeutic strategy. Created with BioRender.com.

Glucose metabolism, including glycolysis, the TCA cycle, and the PPP, constitutes a central energy-generating network within cells. During metabolic reprogramming, macrophages undergo a shift known as the Warburg effect (57), or aerobic glycolysis, wherein they preferentially rely on glycolysis rather than OXPHOS, even under normoxic conditions (58). Under physiological conditions, macrophages predominantly utilize OXPHOS to meet their energetic demands. In contrast, M2-like macrophages adopt a dual metabolic strategy that integrates OXPHOS with fatty acid oxidation, a configuration that maintains energy production while limiting tissue injury caused by excessive inflammation (59, 60). Multiple metabolic pathways influence macrophage behavior. However, this section focuses on the extensively characterized roles of glycolysis and the TCA cycle in regulating macrophage function during ALI progression. These pathways have been most consistently implicated in disease-related metabolic remodeling (**Figure 2**).

### 2.1 Glycolysis and glycolytic enzymes

#### 2.1.1 Activation of glycolysis in macrophages

Glycolysis, the metabolic pathway that converts glucose into pyruvate and subsequently lactate, serves as a key energy-generating process, particularly under anaerobic conditions. Clinical and experimental evidence indicates that lipopolysaccharide (LPS) stimulation markedly enhances glycolytic flux in macrophages, thereby promoting the production of pro-inflammatory cytokines and aggravating lung injury. In a mouse model, Zhong WJ et al. (61) further demonstrated that increases in pro-inflammatory mediators such as pro-IL-1β and TNF-α occur prior to glycolytic activation, supporting the notion that inflammation can initiate metabolic reprogramming. Transcriptional and metabolic profiling of human sepsis patients has revealed that the shift from oxidative phosphorylation to aerobic glycolysis is critical for initial host defense activation, implicating metabolic processes might represent a therapeutic target in sepsis (62).

6-phosphofructo-2-kinase/fructose-2,6-biphosphatase 3 (PFKFB3), a key glycolytic enzyme, is markedly upregulated in macrophages following LPS stimulation, resulting in enhanced glycolytic flux and elevated production of pro-inflammatory cytokines, thereby promoting sepsis progression and exacerbating lung injury and inflammation (63, 64). Beyond its role in glycolysis, PFKFB3 also participates in NLRP3 inflammasome activation (65), NOX4-dependent ROS generation (66), zinc finger and homeobox protein Zhx2 activation (67), and PKM2/HIF-1α signaling (68), with evidence from human macrophage models further supporting its involvement in the PKM2/HIF-1α axis (69). Collectively, these pathways contribute to inflammatory amplification during sepsis. Studies in murine models have shown that myeloid-specific *Pfkfb3* deficiency attenuate pulmonary edema and cardiac dysfunction by suppressing LPS-induced glycolytic flux and pro-inflammatory gene expression; these findings have been validated in both bone marrow-derived macrophages (BMDMs) and pulmonary macrophages (70). Consistently, analysis of serum samples from human patients has revealed that apelin-13, an endogenous ligand for the angiotensin type 1 receptor-associated protein, reduces inflammatory responses, and experimental studies further demonstrate that apelin-13 mitigates acute lung injury by inhibiting PFKFB3-mediated glycolysis in macrophages (66). The natural compound micheliolide (MCL) attenuates sepsis-induced ALI by suppressing PFKFB3-driven glycolysis and M1 polarization, as demonstrated in LPS+IFN-γ-stimulated macrophages and a CLP-induced murine sepsis model (71). These findings indicate that targeting PFKFB3-driven glycolytic metabolism represents a promising strategy for mitigating sepsis-induced organ damage.

Parallel to PFKFB3, another key glycolytic enzyme, pyruvate kinase M2 (PKM2), plays a critical role in macrophage glycolytic reprogramming. As a central mediator of LPS-induced glycolysis (72), PKM2 not only promotes inflammasome activation and the subsequent release of IL-1β, IL-18, and high mobility group box protein B1 (HMGB1), collectively driving systemic inflammatory response syndrome (SIRS) (73), but also directly modulates HIF-1α activity (68). Notably, the flavonoid cynaroside can partially disrupt the PKM2/HIF-1α interaction, thereby altering the transcription of downstream glycolytic and inflammatory genes, facilitating the transition from an M1-like to an M2-like phenotype (61). In addition, protein 4.1R interacts with PKM2 to modulate Toll-like receptor 4 (TLR4) signaling. This interaction suppresses activation of the AKT/HIF-1α pathway and negatively regulates LPS-induced M1 polarization by attenuating HIF-1α-dependent glycolysis (74).

HIF-1α functions as a master transcriptional regulator that induces the expression of glycolytic genes (HK1, PHK1, LDH) as well as pro-inflammatory cytokines (75). Its activity is governed by multiple upstream signals that either promote or restrain its activation. On the activating side, Akt–mTOR-mediated upregulation of HIF-1α markedly enhances glycolytic activation in macrophages (76, 77). Additionally, the E3 ubiquitin ligase MDM2 promotes HIF-1α activation through SPSB2 degradation and subsequent iNOS stabilization, leading to NO-mediated S-nitrosylation of HIF-1α. Notably, MDM2 deficiency protects against LPS-induced endotoxemia but exacerbates mortality in polymicrobial sepsis (78). A recent study further demonstrated that fibroblast growth factor 13 (FGF13), which is significantly downregulated in endothelial cells and macrophages in the lungs of septic patients and mice. It acts as a scaffold protein to promote TAK1/MEK/ERK signaling and HIF-1α-driven aerobic glycolysis, thereby accelerating septic lung injury (79). On the inhibitory side, the short-chain fatty acid acetate (SCFAs) suppresses HIF-1α-dependent glycolysis. Elevated in septic patients and mice, exogenous acetate attenuates glycolytic inflammation via ACSS2-mediated histone acetylation, thereby protecting against sepsis (80). Downstream of these regulatory networks, conditional knockout of HIF-1α in macrophages increases oxygen consumption, reduces inflammatory cytokine and lactate production, and lowers endotoxin-induced mortality, confirming its central role in glycolytic inflammation (81). Building on these mechanistic insights, BMSC-derived exosomes have been shown to inhibit glycolysis in MH-S mouse alveolar macrophages (82) and like recombinant thrombomodulin (rTM) (83), both attenuate macrophage glycolysis via HIF-1α inhibition, thereby ameliorating sepsis-induced lung injury.

Beyond these canonical activation pathways, recent studies have uncovered additional layers of glycolytic regulation in septic macrophages, spanning intercellular communication, endogenous checkpoints, and microenvironmental sensing. With respect to cell-to-cell signaling, endothelial cell-derived CCL7 engages CCR1 on macrophages to activate KAT2A-mediated STAT1 succinylation, which enhances glycolytic gene transcription and drives M1 polarization, thereby exacerbating S-ALI; the therapeutic relevance of this axis is underscored by attenuated lung inflammation and improved survival in Ccr1-deficient mice (84). A parallel mode of communication involves exosomal transfer, whereby type II alveolar epithelial cell-derived Rmrp stabilizes ZFP36 in alveolar macrophages, accelerating Pfkfb3 mRNA decay to impair glycolysis and promote immune tolerance during sepsis-induced immunosuppression. Clinically, exosomal Rmrp levels correlate with alveolar macrophage dysfunction and patient prognosis (85). Opposing these activating cues, endogenous mechanisms restrain excessive glycolytic flux. Interleukin 1 receptor 2 (IL1R2) directly binds and inhibits enolase 1 (ENO1), suppressing glycolysis-driven gasdermin D (GSDMD)-dependent pyroptosis and inflammation; accordingly, IL1R2-deficient mice display heightened sepsis susceptibility, whereas ENO1 inhibition improves outcomes, establishing the IL1R2-ENO1 axis as a critical glycolytic checkpoint (86). In addition, the innate immune sensor Z-DNA binding protein 1 (ZBP1) links glycolytic reprogramming to mitochondrial damage and pyroptosis in septic ALI. ZBP1 deficiency in macrophages reduces mitochondrial injury and suppresses glycolysis, thereby limiting NLRP3 inflammasome-mediated pyroptosis and attenuating macrophage-endothelial inflammatory crosstalk (87). Glycolytic regulation also extends to epigenetic and mechanical domains. Fibroblast growth factor 15 (FGF15), via FGFR4-NF2-Hippo signaling, suppresses glycolysis and lactate production, and the consequent reduction in lactate attenuates histone H3K18 lactylation to decrease Irf7 expression and restrain M1 polarization; rFGF15-treated macrophages alleviate multi-organ inflammation and improve survival in septic mice (88). In the mechanical realm, the mechanosensitive cation channel TRPV4 promotes GLUT1-mediated glucose uptake and glycolysis, facilitating phagolysosome maturation and bacterial clearance to limit lung injury. Notably, elevated bronchoalveolar lavage fluid lactate in septic patients underscores the clinical relevance of pulmonary glycolysis in human sepsis (89). Finally, the transcription factor FoxO1 regulates glycolytic and inflammatory responses in tissue-resident macrophages, and its myeloid-specific deletion alleviates sepsis-induced organ injury (90).

Collectively, these findings demonstrate that glycolytic regulation in septic macrophages operates through a multilayered network encompassing canonical enzymatic pathways, HIF-1α-mediated transcriptional control, intercellular communication, endogenous checkpoints, and mechanical sensing. The convergence of these diverse regulatory mechanisms on glycolysis underscores its dual role as both a metabolic driver of inflammatory macrophage function and a promising therapeutic target in sepsis. Going forward, strategies aimed at modulating macrophage glycolytic flux may offer effective avenues for mitigating sepsis-induced organ injury.

#### 2.1.2 Potential therapeutic approaches targeting glycolysis

Given the central role of macrophage glycolysis in sepsis pathogenesis, targeted modulation of this pathway offers substantial therapeutic potential. Several natural compounds have demonstrated notable efficacy in regulating macrophage metabolism. For example, rhein (RH), a bioactive component abundant in rhubarb, suppresses glycolysis in macrophages and thereby mitigates their excessive activation (91). Likewise, eriocitrin (ERI), a flavonoid compound, attenuates glycolytic activity by inhibiting MAPK signaling in peritoneal macrophages (92). Both agents effectively alleviate lung injury in septic mice, underscoring the therapeutic promise of natural products in inflammatory diseases. Clinical samples from ALI patients and LPS-induced murine models reveal marked elevations in serum lactate along with upregulated expression of key glycolytic regulators, including HK2, PKM2, and HIF-1α. In contrast, treatment with the glycolytic inhibitor 2-deoxy-D-glucose (2-DG) significantly reduces pro-inflammatory factor expression (61). Similarly, the pharmacological inhibitor 3PO improves outcomes in sepsis-induced ALI by reducing mice lung inflammation and ameliorating histopathological damage (93). Given the limitations of existing HK2 inhibitors, a CRISPR/Cas9-mediated HK2 downregulation strategy delivered via an aerosolized core-shell liposomal nanoplatform (CSNs) targeting pulmonary macrophages has been developed. In an LPS-induced ALI mouse model, inhaled CSN-mCas9/gHK2 effectively attenuated the proinflammatory microenvironment and reprogrammed glucose metabolism in the lung (94).

As a member of the PFK family, PFKFB3 plays a dynamic role in regulating glycolytic flux, particularly in macrophages, where its expression directly influences glucose uptake efficiency and ATP production. Inhibition of PFKFB3 not only reduces lactate accumulation but also modulates the balance between M1 and M2 macrophage polarization. Hypoxia, a hallmark of sepsis, further amplifies PFKFB3 activity through PI3K/Akt/mTOR and HIF-1α signaling pathways, thereby driving excessive lactate production and intensifying pro-inflammatory responses (69). This metabolic dysregulation presents a promising therapeutic target, and several pharmacological strategies have been explored. For instance, inhibition of p38 MAPK markedly decreases PFKFB3 expression and lactate production in BMDMs (95). Similarly, WIN55212-2 (WIN) attenuates M1 polarization and glycolysis in LPS-induced alveolar macrophages via the miR-29b-3p/FOXO3/PFKFB3 axis, thereby alleviating sepsis-induced ALI (96). Natural products have also shown efficacy in targeting PFKFB3. The flavonoid cynaroside mitigates sepsis-induced liver injury by suppressing PFKFB3-driven glycolytic metabolism and preventing macrophage polarization toward the M1 phenotype (68). Likewise, the ethanol extract of Physalis angulata fruit (EPAF) modulates PFKFB3 acetylation and phosphorylation to inhibit glycolysis and M1 polarization, thereby alleviating LPS-induced lung injury (97). Beyond PFKFB3, the liver-specific PFK isozyme phosphofructokinase, liver type (PFKL) has emerged as another regulatory node. TGF-β uniquely regulates PFKL, enhancing its expression to promote glycolysis while simultaneously suppressing pro-inflammatory cytokine production (98). The flavonoid orientin directly binds and inhibits PFKL, suppressing glycolytic flux and inflammatory cytokine production in septic macrophages. Notably, macrophage-specific PFKL overexpression abrogates these protective effects, confirming PFKL as its critical target (99). Together, these findings suggest that targeting PFK family enzymes represents a promising therapeutic avenue, with TGF-β emerging as an important regulator of macrophage metabolism and a potential target in sepsis.

Modulation of PKM2 represents another promising therapeutic strategy. Several plant-derived compounds exert anti-inflammatory effects by targeting PKM2-mediated metabolic pathways. Deoxyelephantopin (DET) reduces PKM2 mRNA and protein expression and inhibits its nuclear translocation in LPS-stimulated macrophages, thereby decreasing aerobic glycolysis and the release of inflammatory mediators such as IL-1β and HMGB1. These effects ultimately lower mortality in endotoxemia and sepsis models (100). Lycium barbarum polysaccharide (LBP) also suppresses LPS-induced inflammation by modulating PKM2 ubiquitination, altering macrophage RAW264.7 glycolysis, and regulating M1 polarization in ALI (101). Norisoboldine also decreases M1 macrophage polarization and enhances M2 polarization through regulation of the PKM2/HIF-1α/PGC-1α signaling pathway, thereby ameliorating LPS-induced ALI (102). Beyond plant-derived compounds, the clinically used sedative dexmedetomidine (DEX) has been shown to attenuate sepsis-induced lung injury by suppressing PKM2 Y105 phosphorylation and nuclear translocation, disrupting glycolytic flux and reducing M1 polarization (103). More recently, the natural saponin platycodin D3 (PD3) was identified as a direct PKM2-binding compound. PD3 stabilizes PKM2 and disrupts the PKM2-NLRP3 inflammasome axis, restoring metabolic homeostasis and alleviating sepsis-induced lung injury (104). In addition to its role in acute inflammation, PKM2 has also been implicated in tissue repair. Macrophage-specific PKM2 deletion enhances mucosal healing in murine colitis by promoting reparative macrophage differentiation while simultaneously suppressing tumorigenesis. Cross-species analysis further identified human STAB1^+^ macrophages as a functionally analogous population associated with ulcerative colitis (UC) remission. Thus, PKM2 modulation may offer dual benefits of inflammation resolution and regenerative repair in sepsis and other inflammatory disorders (105).

The PKM2–HIF-1α axis represents a central metabolic–inflammatory signaling hub, as HIF-1α serves as a master regulator of glycolytic reprogramming. Several pharmacological strategies have been developed to target this axis. Xijiao Dihuang decoction (XJDHT), a traditional Chinese medicine, inhibit the TLR4–HIF-1α signaling pathway, suppress aerobic glycolysis, and improve survival in rat sepsis models (106). TLR4 also coordinates macrophage-endothelial crosstalk and regulates cholesterol efflux via Abca1 in septic S-ALI, further highlighting its multifaceted role in disease pathogenesis (107). A newly synthesized HIF-1α inhibitor, N-phenethyl-5-phenylpicolinamide (N5P), modulates glycolysis and LPS-induced inflammatory responses in RAW264.7 macrophages and rat primary alveolar macrophages via HIF-1α–dependent mechanisms (108). Natural products have also shown efficacy in disrupting this axis. Epigallocatechin gallate (EGCG), a major green tea catechin, suppresses HIF-1α expression by inhibiting EGFR phosphorylation, thereby reducing HK2 and PKM2 expression and attenuating sepsis-induced lung injury (109). Additionally, suppressor of cytokine signaling 1 (SOCS1) negatively regulates cytokine and Toll-like receptor signaling by downregulating the STAT3/HIF-1α/glycolysis axis. Through this mechanism, SOCS1 functions as an important inhibitor of macrophage glucose metabolism reprogramming, limiting excessive inflammatory responses and protecting against sepsis-induced organ injury in mice (110).

Beyond metabolic enzymes, several additional regulatory pathways offer promising therapeutic avenues for S-ALI. AMP-activated protein kinase (AMPK) functions as a negative regulator of macrophage glucose metabolism reprogramming. Its deficiency exacerbates LPS-induced, PKM2-dependent aerobic glycolysis and increases the release of HMGB1, effects reversed by the PKM2 inhibitor shikonin (111). Conversely, AMPK activation confers protection. Plasma growth differentiation factor 15 (GDF15) correlates with disease severity in septic patients. In experimental sepsis models, GDF15 activates AMPK to suppress glycolysis and MAPK/NF-κB signaling in alveolar macrophages, thereby alleviating lung injury (112). Cryptotanshinone similarly activates AMPK, inhibiting M1 and promoting M2 polarization by counteracting LPS-induced metabolic reprogramming (113). Sphingosine-1-phosphate (S1P) signaling also emerges as a key regulatory node. As a major product of sphingolipid catabolism, S1P binds to its receptor S1PR3, which is upregulated in LPS-activated macrophages and septic lungs, to activate pro-glycolytic pathways involving HIF-1α, HK2, and PFKFB3. Inhibition of sphingosine kinase 1 (SphK1) with PF-543 modulates macrophage polarization and suppresses the Warburg effect via a SphK1/S1PR3-dependent mechanism, alleviating excessive inflammation and multi-organ failure (114). Further supporting the relevance of this axis, Spinster homolog 2 (Spns2), the primary S1P transporter, regulates inflammation through the lactate–ROS pathway. Loss of Spns2 enhances glycolysis and lactate accumulation in macrophages, whereas augmentating of Spns2/S1P signaling restores immune homeostasis and prevents both early hyperinflammation and late immunosuppression in sepsis (115). Innovative delivery platforms have expanded the therapeutic arsenal beyond single-target approaches. A self-assembled nanoparticle, LDO, integrates an itaconate derivative with the glycolysis inhibitor lonidamine, concurrently suppressing glycolysis and activating the itaconate-STING axis to reprograms macrophage polarization, attenuates cytokine storms, and improves survival (116). Engineered exosome-based delivery systems offer another: fibroblast growth factor 21 (FGF21)-loaded M2 macrophage-derived exosomes (FGF21-M2-Exos) achieve sustained pulmonary delivery of the short-lived metabolic regulator FGF21. In sepsis-induced ALI models, these exosomes inhibit glycolysis, promote M2 polarization, and reduce apoptosis, alleviating lung injury (117). pH-responsive nanoparticles represent a complementary nanotherapeutic approach. BAY-876@NPs, which release the GLUT1 inhibitor BAY-876 selectively in acidic inflammatory microenvironments, suppressing glucose uptake and glycolysis to promote M2 polarization and improve survival in septic mice (118). A biomimetic nanoplatform (RM@TNT) further expands the toolkit by delivering a BMAL1 agonist via alveolar macrophage membrane-derived nanovesicles and ROS-responsive liposomes, thereby suppressing PFKFB3-driven glycolysis, attenuating inflammation and improving survival in SA-ARDS mice (119). Notably, the pulmonary niche imposes metabolic constraints that limit therapeutic efficacy. Svedberg et al. showed that alveolar macrophages exhibit impaired glycolysis and IL-4 hyporesponsiveness, defects reversed upon removal from the lung environment in a glycolysis-dependent manner (120). This finding suggests that overcoming tissue-specific metabolic barriers may enhance therapeutic outcomes in the lung.

### 2.2 The TCA cycle and its products

#### 2.2.1 The tricarboxylic acid cycle and its products

The TCA cycle is essential for generating the large quantities of ATP required for normal physiological function and serves as a central metabolic hub that integrates glucose, lipid, and amino acid metabolism. Distinct metabolic profiles characterize polarized macrophages: M1-like macrophages rely primarily on aerobic glycolysis, fatty acid synthesis, and a truncated TCA cycle, whereas M2-like macrophages depend on FAO and a fully oxidized TCA cycle (121). This metabolic divergence underpins their functional specialization during inflammation. LPS, a classical pro-inflammatory stimulus, enhances glycolytic flux while concurrently suppressing TCA cycle activity and OXPHOS. Despite this inhibition, LPS-activated macrophages exhibit notable accumulation of several TCA intermediates, including citrate, succinate, malate, and fumarate (122), which contribute to downstream inflammatory responses, particularly the induction of IL-1β. Notably, citrate synthase (CS), the rate-limiting enzyme initiating the TCA cycle, is significantly downregulated in septic patients and positively correlates with lung function. CS promotes TCA cycle activity in pulmonary macrophages, alleviating mitochondrial damage and protecting against sepsis-induced lung injury (123).

Activation of macrophages by interferon-γ (IFN-γ) and LPS leads to truncation of the TCA cycle at the levels of isocitrate dehydrogenase (IDH) and succinate dehydrogenase (SDH), resulting in the accumulation of key intermediates such as succinate and citrate. Citrate serves as the precursor for the macrophage-specific metabolite itaconate, a hallmark of IFN-γ/LPS-polarized macrophages, and its levels increase markedly upon LPS stimulation (124). Studies in both mouse and human macrophages have demonstrated that itaconate levels rise substantially following LPS exposure and that this metabolite represents the first metabolite directly linked to the antimicrobial activity of pro-inflammatory macrophages (125, 126). Moreover, itaconate exerts therapeutic effects in S-ALI by inhibiting ferroptosis, a finding consistently observed both in THP-1 cells and in Nrf2-knockout mice (127). Functionally, itaconate acts as an endogenous inhibitor of SDH (128). Consistent with this role, LPS-stimulated IRG1^–/–^ mouse bone marrow-derived macrophages, which are unable to synthesize itaconate, fail to accumulate succinate, demonstrating that itaconate is a critical driver of succinate accumulation in activated macrophages (129, 130). Beyond its local regulation within the TCA cycle, itaconate production is also governed by upstream metabolic inputs. Glutamine metabolism regulates itaconate production through TFEB-dependent IRG1 transcription. Glutamine deprivation impairs this pathway, reducing itaconate levels and enhancing NLRP3 inflammasome activation and pyroptosis. These effects are reversed by α-ketoglutarate or exogenous itaconate, and glutamine supplementation protects against intestinal inflammation in septic mice (131).

Mitochondrial dysfunction drives enhanced glycolytic activity by impairing the TCA cycle and reducing OXPHOS. Virga et al. demonstrated that miR-210 suppresses mitochondrial respiration while promoting glycolysis. Its knockout in the hematopoietic lineage or monocytes/macrophages shifts metabolism toward an anti-inflammatory phenotype, mitigating the severity of endotoxemia, bacteremia, sepsis, and parasitic infections. Clinically, elevated circulating monocyte miR-210 correlates with sepsis incidence, and serum miR-210 is associated with sepsis mortality (132). In another study, Li et al. used phagocytes (including macrophages) from sepsis patients and an LPS-induced mouse sepsis model to identified mitochondrial STAT3 as a key regulator promoting the nuclear translocation of NF-κB in macrophages and exacerbates LPS-induced sepsis. STAT3 also redirects macrophage metabolism from glucose utilization toward fatty acid oxidation via USP50-mediated stabilization of CPT1A. These detrimental effects are reversed by curcumin (133). In contrast, the mitochondrial antioxidant enzyme peroxiredoxin 3 (PRDX3) promotes the transition from M1-like to M2-like macrophages. PRDX3 enhances mitochondrial recovery after injury by reducing glycolytic flux and increasing TCA cycle activity (134). Beyond its role in glycolytic, Spns2 also governs mitochondrial respiration through PGE2. Studies in Spns2 knockout rats, macrophage-specific Spns2-deficient mice, and THP-1 cells demonstrate that Spns2/S1P signaling suppresses PGE2 production. This maintains malate-aspartate shuttle activity and mitochondrial respiration. Spns2 deficiency elevates PGE2, impairing mitochondrial function and driving lactate-ROS-mediated early hyperinflammation and subsequent immunosuppression (135). Mitochondrial dynamics in alveolar macrophages are further regulated by the SIRT3-OPA1 axis. SIRT3 deacetylates OPA1 at lysine 792, preventing its self-cleavage and maintaining mitochondrial fusion. This restrains M1 polarization and protects alveolar epithelial barrier function in sepsis-induced ALI (136). These findings highlight mitochondrial function as a critical determinant of macrophage inflammatory responses and suggest that targeting mitochondrial regulatory pathways may provide novel therapeutic strategies for macrophage-driven inflammatory diseases.

#### 2.2.2 Potential therapeutic approaches targeting TCA cycle intermediates

Targeting TCA cycle intermediates offers a promising strategy for modulating macrophage polarization under inflammatory conditions. During LPS-induced M1 polarization, accumulated succinate stabilizes HIF-1α by inhibiting prolyl hydroxylase (PHD) activity, thereby enhancing glycolytic flux and amplifying inflammatory responses. This process can be attenuated by 2-DG, with IL-1β serving as a key downstream target (137). In contrast, supplementation with α-ketoglutarate (α-KG) exerts dual regulatory effects. α-KG suppresses M1 polarization by inhibiting the mammalian target of rapamycin complex 1 (mTORC1)/p70 ribosomal protein S6 kinase (p70S6K) signaling pathway and promotes M2 polarization by augmenting peroxisome proliferator-activated receptor γ (PPARγ) nuclear translocation and fatty acid metabolic genes. These effects have been validated both in MH-S cells (a murine alveolar macrophage cell line) *in vitro* and in mouse models of acute lung injury (138). The therapeutic relevance of TCA cycle modulation extends beyond metabolic intermediates. The widely used synthetic glucocorticoid dexamethasone (DEX) promotes TCA cycle rewiring in the livers of septic mice. DEX also inhibits JAK1/STAT3 signaling, reducing macrophage infiltration and inflammatory cytokine production to alleviate organ injury and improve survival. Although these findings derive from a liver injury model, they may hold relevance for sepsis-associated lung injury (139). Returning to α-KG, this metabolite also exerts anti-inflammatory effects through a distinct epitranscriptomic mechanism. α-KG upregulates the m6A demethylase ALKBH5, reducing m6A methylation levels in macrophages. This modulates the expression of scavenger receptor SR-A5, a pattern recognition receptor with vital anti-inflammatory functions, and alleviates sepsis-induced inflammation and tissue damage (140).

During inflammation, the transition from M1-like to M2-like macrophages is accompanied by a metabolic shift from aerobic glycolysis toward glucose-derived OXPHOS (62, 141, 142). At the peak of inflammation in sepsis, immune cells and organ cells rely predominantly on glycolysis rather than OXPHOS to meet acute energy demands. However, excessive lactate generated by sustained glycolysis promotes immune cell death and immune suppression, while persistent mitochondrial dysfunction in the late stage impairs OXPHOS restoration and perpetuates organ damage. Uncoupling protein 2 (UCP2), a key member of the mitochondrial uncoupling protein family, is widely expressed in hepatic and immune cells, and regulates the balance between OXPHOS and glycolysis by modulating mitochondrial respiration. UCP2 overexpression attenuates the Warburg effect and mitigates damage caused by excessive glycolytic flux, thereby exerting beneficial effects during sepsis (143), whereas UCP2 silencing exacerbates both (144). However, the differential expression of UCP2 during sepsis can elicit distinct and even opposing biological effects. Clinically, a study by Jiang et al. (145) blood cell samples from 69 sepsis patients, 87 severely ill sepsis patients, and 69 healthy controls revealed significantly higher UCP2 mRNA and protein levels in sepsis patients before treatment. Notably, patients with elevated UCP2 levels at admission exhibited higher mortality, a finding corroborated by two additional prospective clinical studies (146, 147). In striking contrast, multiple experimental studies have consistently demonstrated protective effects of UCP2 overexpression. In a model of sepsis-associated acute kidney injury, Ding et al. (148) found that although UCP2 expression was increased in septic mice, UCP2 overexpression actually alleviated mitochondrial dysfunction and protected HK-2 cells from endotoxin-induced injury. Similarly, Ji et al. (149) reported that UCP2 upregulation attenuates the Warburg effect and alleviates LPS-induced mitochondrial dysfunction in renal tubular epithelial cells. Chen et al. (150) further reported that UCP2 overexpression reverses septic myocardial cell damage, reduces cardiac enzyme release and inflammatory cytokine secretion, and suppresses ROS production. The protective effects of UCP2 extend to human primary macrophages, where its overexpression similarly attenuates glycolysis and promotes M1-to-M2 polarization (151).This apparent discrepancy may be explained by the fact that the biological effects of UCP2 during sepsis vary depending on the stage of disease progression and the specific metabolic microenvironment. The elevated UCP2 levels observed in clinical settings may reflect a compensatory response of the host to metabolic dysregulation. However, the magnitude of endogenous UCP2 upregulation may be insufficient to fully counteract severe mitochondrial damage and metabolic imbalance. In contrast, experimental overexpression further amplifies this protective mechanism, thereby conferring clear therapeutic benefits. Collectively, UCP2 participates in both metabolic and immune processes. Although its role presents a complex dual aspect in clinical versus basic research settings, these findings suggest that UCP2 may serve as a promising diagnostic biomarker for sepsis, with potential utility in early detection and prognosis evaluation.

Overexpression of UCP2 reduces macrophage glycolysis while enhancing OXPHOS, thereby decreasing ROS production and promoting the transition from an M1 to an M2 phenotype (151). The relationship between ROS and macrophage polarization in sepsis is complex and context-dependent. On one hand, ROS production is essential for effective host defense: Parkinson disease (autosomal recessive, early onset) 7 (Park7) has been shown to promote NADPH oxidase-dependent ROS generation by binding to p47phox, thereby supporting macrophage polarization and bacterial clearance; Park7 deficiency impairs ROS production and increases mortality in murine sepsis models (152). On the other hand, excessive or uncontrolled ROS production contributes to tissue damage and inflammation. Accordingly, therapeutic strategies that mitigate oxidative stress, such as IL-10 administration following TLR4 and TNFR1 neutralization, have been shown to promote M2 polarization, enhance antioxidant enzyme activities, and confer protection against LPS-induced sepsis (153). During sepsis, family with sequence similarity 96 member A (FAM96A) has been shown to mediate an immunometabolic shift from OXPHOS to glycolysis. This evolutionarily conserved protein is highly expressed in the immune system and is involved in cytosolic iron assembly and tumor suppression. The metabolic shift it mediates is accompanied by increased ROS generation and glucose uptake (154). Accumulated succinate during immunometabolic reprogramming further amplifies mitochondrial ROS production through enhanced SDH activity, establishing a pro-inflammatory feedback loop that reinforces the glycolytic shift (155). Interestingly, Tsai et al. (156) reported that in the human monocyte cell line, IL-25, a Th2 cytokine, induces ROS production and increases mitochondrial respiratory chain complex activity, leading to AMPK activation and subsequent M2 macrophage polarization.

## 3 Lipid metabolism

**Figure 3:**
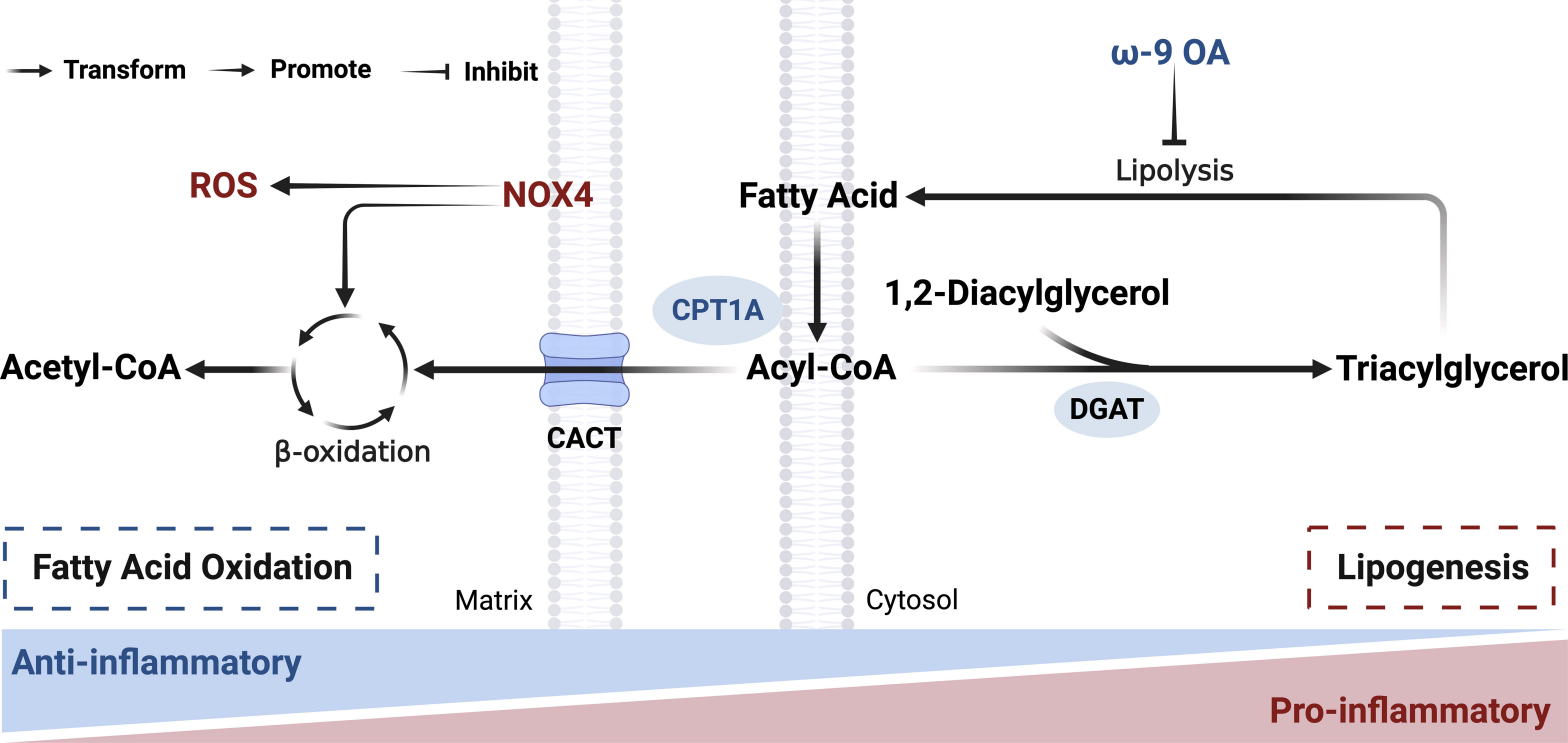
Reprogramming of Lipid metabolism during macrophage polarization. Pro-inflammatory macrophages exhibit a metabolic profile characterized by enhanced lipogenesis and triglyceride synthesis, a process driven in part by diacylglycerol acyltransferase (DGAT) in the cytosol, while ω-9 oleic acid (OA) suppresses lipolysis. In contrast, anti-inflammatory macrophages rely predominantly on mitochondrial fatty acid oxidation (FAO), a process facilitated by the rate-limiting enzyme carnitine palmitoyltransferase 1A (CPT1A) to import fatty acids into the matrix. NADPH oxidase 4 (NOX4) enhances mitochondrial ROS production and FAO. Created with BioRender.com.

Lipids and their metabolites play essential roles in macrophage biology by regulating cytokine expression and modulating diverse signaling pathways. Lipid metabolism includes lipogenesis, fatty acid β-oxidation (FAO), lipolysis, and lipid uptake and transport. FAO is a particularly important metabolic pathway in macrophages during sepsis, especially under conditions of dysregulated inflammatory activation (157). M1-like macrophages rely on lipogenesis to support lipid accumulation and phagocytic activity, whereas M2-like macrophages primarily utilize FAO to generate energy from fatty acids (158, 159). These contrasting metabolic preferences, FAS-driven pathways in pro-inflammatory M1-like macrophages versus FAO-driven pathways in immunosuppressive M2-like macrophages, highlight the central role of fatty acid metabolism in sepsis pathophysiology (**Figure 3**). Therapeutically targeting these lipid metabolic pathways offers significant potential to reprogram macrophage polarization, reduce excessive inflammation, and improve clinical outcomes in sepsis and related inflammatory disorders.

### 3.1 Clinical and laboratory insights into fatty acid metabolism

Clinical and laboratory evidence demonstrates pronounced disruptions in fatty acid metabolism during sepsis. At the clinical level, metabolomic analyses have identified altered fatty acid profiles as potential biomarkers for disease prognosis. In a cohort of approximately 150 sepsis patients, distinct alterations in fatty acid metabolism were observed between survivors and non-survivors. Sepsis non-survivors exhibited elevated plasma levels of multiple carnitine esters and free fatty acids, along with decreased levels of several fatty acid transport proteins. These findings suggest a profound defect in fatty acid β-oxidation at the level of the carnitine shuttle that was absent in survivors (160). Further supporting the clinical relevance of lipid metabolism, supplementation studies have shown that immuno-modulating diets (IMDs) enriched with ω-3 fatty acids and vitamin E reduce secondary infection risk and shorten ICU length of stay in both adult and children (161–164). In parallel with these clinical observations, laboratory studies have elucidated the cellular mechanisms underlying sepsis-associated lipid dysregulation. In RAW264.7 cells and peritoneal macrophages, LPS stimulation promotes triglyceride accumulation by increasing fatty acid uptake, enhancing glucose-derived lipid incorporation, upregulating triglyceride synthesis, and suppressing lipolysis (165). Consistently, elevated citrate and fatty acid levels in BMDMs during acute inflammation indicate a shift toward fatty acid synthesis (124). Importantly, these metabolic alterations are not uniform across macrophage phenotypes. M1-like macrophages exhibit pro-lipogenic reprogramming, whereas IL-4–stimulated M2-like macrophages show enhanced FAO and suppressed glycolysis (166). Huang et al. showed that CD36-mediated triacylglycerol uptake and lysosomal acid lipase-dependent lipolysis represent one source of fatty acids that support this metabolic program (167). Beyond lipid uptake, fatty acid sensing also contributes to metabolic regulation. The short-chain fatty acid receptor FFAR2 regulates lipid peroxidation and ferroptosis in septic macrophages. The transcription factor VDR upregulates FFAR2 by binding to its promoter, increasing ferroptosis-negative regulatory molecules and reducing lipid peroxide accumulation. Activation of this VDR/FFAR2 axis alleviates lung injury in septic mice, highlighting its immunotherapeutic potential (168). This phenotypic specificity of lipid metabolic remodeling highlights the divergent bioenergetic strategies employed by distinct macrophage subsets in sepsis.

Research on key enzymes regulating lipid metabolism further clarifies the metabolic basis of macrophage function in sepsis. The anti-inflammatory phenotype of M2-like macrophages, induced by IL-4/IL-13, depends largely on OXPHOS and FAO and is associated with reduced mTOR signaling (169–171). Carnitine palmitoyl transferase 1A (CPT1A), the rate-limiting enzyme responsible for mitochondrial fatty acid import, plays a critical protective role in this process. In an LPS-induced ALI mouse model, macrophage-specific deletion of CPT1A (Cre^+^CPT1A^f1^/^f1^) exacerbated pulmonary inflammation, enhanced inflammatory cell infiltration, and worsened the response to LPS. This protective effect of CPT1A is partly attributed to its ability to sustain IL-10 expression in lung macrophages and to promote IL-10/rIL-10 signaling, which together drive the metabolic shift from glycolysis toward FAO and facilitate M2 polarization (172). FAO activity is also subject to negative regulation during sepsis. The lipid-associated macrophage marker TREM2 acts as a suppressor of FAO. TREM2 is markedly upregulated in septic patients and correlates with disease severity. Its macrophage-specific knockout enhances BTK-mediated FAO, reduces triglyceride accumulation, and improves survival in septic mice. Mechanistically, TREM2 interacts with the phosphatase SHP1 to inhibit BTK-mediated FAO, identifying TREM2 as a critical negative regulator and a potential therapeutic target (173). Another layer of FAO regulation involves estrogen receptor β (ERβ), which links FAO to mitochondrial dysfunction and pyroptosis. ERβ expression is significantly reduced in septic patients and negatively correlates with disease severity. ERβ deficiency enhances FAO, increasing acetyl-CoA levels and promoting Stoml2 acetylation at K221. This triggers mitochondrial dysfunction and macrophage pyroptosis, impairing bacterial clearance and increasing sepsis susceptibility (174). Additionally, NADPH oxidase 4 (NOX4) enhances mitochondrial ROS production and FAO, and its inhibition suppresses NLRP3 inflammasome activation and improves survival in infectious disease models (66).

### 3.2 Potential therapeutic approaches targeting lipid metabolism

Therapeutic strategies targeting lipid metabolism show considerable promise for mitigating sepsis-induced lung injury. These interventions may act on metabolites or key metabolic enzymes to modulate macrophage polarization.

At the metabolite level, supplementation with ω-3 fatty acids exemplifies a clinically validated approach. Immunomodulatory diets enriched with ω-3 fatty acids and vitamin E reduce secondary infection risk and shorten ICU length of stay in both adult and pediatric septic patients. These benefits are likely mediated through inhibition of NF-κB and caspase-1, thereby decreasing IL-1β production (161–164). Likewise, administration of ω-9 oleic acid (OA) reduces non-esterified fatty acid (NEFA) levels, increases the expression of CPT1A, UCP2, and AMPK, lowers ROS production, and alleviates sepsis symptoms in murine models (175). The natural compound esculetin (ELT) also rebalances macrophage polarization in S-ALI. ELT inhibits glycolysis in M1 macrophages and enhance fatty acid β-oxidation in M2 macrophages, as validated in a CLP-induced septic rat model and *in vitro* assays using the glycolysis inhibitor 2-DG and fatty acid β-oxidation inhibitor etomoxir (176).

Despite these promising results, emerging evidence suggests that ω-3 fatty acids are not universally protective in sepsis. In a mouse CLP model, ω-3 supplementation preceding surgery unexpectedly worsened survival without affecting inflammatory cytokine levels. A possible explanation lies in the downregulation of the ω-3 receptor GPR120 during inflammation, which may limit ω-3 efficacy in certain septic contexts (177). These findings caution against indiscriminate ω-3 use and underscore the need for context-specific evaluation of immunonutrition. At the enzymatic level, inhibition of NOX4 represents a targeted strategy. NOX4 inhibition decreases the release of pro-inflammatory cytokines such as IL-1β and IL-18 and improves survival in pneumococcal infection and sepsis models (66).

Despite these encouraging preclinical findings, the translational landscape is complicated by fundamental aspects of macrophage biology. A key unresolved issue concerns the metabolic requirements for M2 polarization and the plasticity of macrophage phenotypes. Studies in murine models have consistently linked M2 polarization to enhanced FAO and oxidative phosphorylation (166, 172). However, the necessity of FAO for M2 activation remains controversial. Pharmacological inhibition of CPT1 with etomoxir does not impair IL-4–induced M2 polarization in either human or murine macrophages, suggesting that FAO may be dispensable, or that compensatory metabolic pathways can sustain M2 function when FAO is blocked (178, 179). This suggests that FAO may be dispensable, or that compensatory pathways sustain M2 function when FAO is blocked. Further complicating therapeutic efforts, Van den Bossche et al. demonstrated that mouse and human M1 macrophages fail to convert into M2 cells upon IL-4 exposure. Nitric oxide-mediated inhibition of mitochondrial oxidative phosphorylation renders these cells metabolically inflexible and resistant to repolarization (179). Notably, M2 macrophages retain greater plasticity and can readily revert to an inflammatory M1 state. Moreover, fundamental species-specific differences exist. Mouse M2 polarization is tightly coupled to FAO upregulation, whereas human macrophages exhibit only modest mitochondrial changes following IL-4 stimulation and do not require FAO for phenotypic switching (178).

These observations have important therapeutic implications. Strategies aimed solely at enhancing FAO may not reprogram inflammatory macrophages in human sepsis, given the metabolic inflexibility of M1 cells and the dispensability of FAO for human M2 polarization. Future therapeutic development should therefore consider combinatorial approaches. These should simultaneously restore mitochondrial function, overcome M1 metabolic rigidity, and account for species-specific differences in immunometabolic regulation. Restoring mitochondrial oxidative capacity, rather than simply augmenting FAO, may represent a more promising avenue for promoting the resolution of inflammation in sepsis-induced lung injury.

## 4 Amino acid metabolism

Amino acid metabolism is essential for numerous cellular processes, providing not only the substrates for protein synthesis but also precursors for the production of branched-chain fatty acids. The catabolism of both endogenous and dietary amino acids contributes to ATP generation and supplies citrate for fatty acid biosynthesis. Furthermore, the carbon skeletons of α-ketoacids, the initial products of transamination, can enter the TCA cycle at multiple points, enabling metabolic integration and flexibility. This versatility ensures that excess cellular amino acids are efficiently utilized as an alternative energy source, supporting cellular function during metabolic stress.

### 4.1 Clinical and laboratory findings on amino acid metabolism

Amino acid metabolism has attracted increasing attention due to its critical role in macrophage polarization and immune regulation. Clinical studies demonstrate profound alterations in circulating amino acid profiles during sepsis. 25 septic patients exhibit elevated plasma levels of 3-methyl-L-histidine, α-aminoadipic acid, α-amino-n-butyric acid, argininosuccinic acid, β-aminoisobutyric acid (BAIBA), carnosine, cystathionine, glutamine, phenylalanine, and proline, while arginine, asparagine, aspartic acid, cystine, glutamic acid, leucine, serine, taurine, and tryptophan are markedly reduced (180). These metabolic shifts likely reflect systemic disturbances in protein catabolism and synthesis under inflammatory stress. Notably, branched-chain amino acids (BCAAs) are significantly lower in sepsis non-survivors than in survivors, indicating that BCAA levels may serve as an early prognostic biomarker for disease severity (181). Further linking amino acid metabolism to immune regulation, ubiquitin regulatory X domain-containing protein 6 (UBXN6) is upregulated in septic patients and negatively correlates with inflammatory gene profiles. Myeloid-specific UBXN6 deficiency exacerbates inflammation, impairs autophagy, and induces metabolic remodeling characterized by enhanced aerobic glycolysis and elevated BCAA levels. Mechanistically, UBXN6 deficiency activates mTOR signaling, reducing TFEB nuclear translocation and lysosomal biogenesis, thereby compromising autophagic clearance (182).

Laboratory investigations further highlight the essential role of amino acid metabolism in regulating macrophage function. Glutamine serves as a versatile metabolic substrate, fueling the TCA cycle, supporting fatty acid synthesis, contributing to ATP production, and facilitating IL-1β secretion in M1-like macrophages. Importantly, glutamine is indispensable for M2 macrophage polarization, where it regulates the expression of the M2 marker CD206 and exerts anti-inflammatory effects. In contrast, M1 macrophages appear less dependent on glutamine, although the underlying mechanisms remain unclear (183). Tryptophan and arginine similarly play key roles in supporting macrophage anabolic growth and proliferation under physiological conditions (184).

During inflammation, arginine metabolism becomes particularly critical, and its availability is tightly regulated at multiple levels (185). Arginine transport systems are dynamically remodeled during monocyte-to-macrophage differentiation. SLC7A7 upregulation serves as a differentiation marker, as demonstrated in human THP-1 cells (186). In activated macrophages, arginine transport is drastically increased via the inducible cationic amino acid transporter CAT2 (SLC7A2). This upregulation is essential for sustaining the high rates of arginine catabolism required for nitric oxide (NO) and polyamine synthesis (187–189). CAT2-deficient macrophages exhibit a 95% reduction in L-arginine uptake and a 92% decrease in NO production upon stimulation (188). Furthermore, CAT2 expression is required for pulmonary fibrotic responses and optimal arginase activity in macrophages (190). Glutamate transport also plays a protective role in S-ALI. The cystine/glutamate antiporter system XC- (xCT subunit) is upregulated in septic mouse lungs and LPS-stimulated macrophages. Its inhibition or knockdown exacerbates lung injury, and clinically, the arterial-venous glutamate gradient correlates with disease severity in ALI/ARDS patients, highlighting its prognostic and therapeutic potential (191). Beyond its role as a substrate, extracellular arginine availability itself functions as a key regulatory signal. Arginine starvation leads to a striking suppression of iNOS protein translation without affecting iNOS mRNA levels or global protein synthesis. This effect is mediated by arginine depletion via arginase activity (192, 193). Notably, this translational control operates independent of the canonical integrated stress response involving eIF2α phosphorylation, indicating alternative, arginine-specific regulatory mechanisms (193). Conversely, arginine supplementation accelerates macrophage polarization by enhancing LPS-induced reactive oxygen species (ROS) production, NO release, and iNOS expression. These effects are mediated through G-protein-coupled receptors and downstream MAPK and phospholipase C signaling (194, 195). The metabolic fate of arginine diverges markedly between macrophage phenotypes. M1-like macrophages use inducible iNOS to convert arginine into NO, which confers potent antimicrobial activity (196–198). In contrast, M2 macrophages employ Arg-1, a canonical M2 marker, to metabolize arginine into ornithine and urea, thereby promoting tissue repair and wound healing (199, 200). This interplay, especially when arginase activity is elevated, can suppress NO production and significantly influence inflammatory outcomes (201). Although amino acid metabolic reprogramming remains less extensively studied than other metabolic pathways, elucidating its regulatory role in macrophage polarization holds substantial promise for advancing the understanding of fundamental immunological mechanisms and for developing novel immunometabolic therapeutic strategies.

### 4.2 Potential therapeutic approaches targeting amino acid metabolism

Emerging therapeutic strategies increasingly focus on modulating amino acid metabolism to influence macrophage polarization and improve sepsis outcomes. At the metabolite level, supplementation with α-aminobutyric acid (AABA), a non-proteinogenic amino acid derived from methionine, threonine, serine, and glycine, represents one promising approach. In mouse BMDMs, AABA inhibits LPS-induced M1 polarization by promoting oxidative phosphorylation and glutamine/arginine metabolism while suppressing glycolysis. Mechanistically, AABA increases H3K27 trimethylation at the promoters of M1-associated inflammatory genes, epigenetically silencing their expression (202). These effects prolonged survival in septic mice and reduced disease severity in murine colitis models. Similarly, β-aminoisobutyric acid (BAIBA), a valine-derived myokine elevated in septic patients, protects against LPS-induced sepsis. Oral BAIBA pretreatment dose-dependently reduces mortality, systemic inflammation, and multi-organ injury in septic mice. *In vitro*, BAIBA suppresses pro-inflammatory macrophage polarization via AMPK pathway activation. Notably, BAIBA co-administration with low-dose dexamethasone provides enhanced therapeutic benefits (203). Asymmetric dimethylarginine (ADMA), an endogenous methylarginine, modulates the NO/ROS balance in activated macrophages by suppressing iNOS expression and NF-κB activation. Its concurrent induction of superoxide formation, however, warrants careful therapeutic calibration. These effects have been demonstrated in both RAW264.7 and MHS macrophage cell lines (194).

At the post-translational level, modulation of STAT3 O-GlcNAcylation offers another therapeutic avenue. In macrophages, O-GlcNAc modification of STAT3 at threonine 717 negatively regulates its phosphorylation and downstream gene expression. The E3 ubiquitin ligase cullin 3 (CUL3) downregulates O-GlcNAc transferase (OGT) via Nrf2-dependent transcriptional control, thereby limiting STAT3 O-GlcNAcylation. Notably, myeloid-specific deletion of Cul3 impaired STAT3 phosphorylation in colonic macrophages and exacerbated colonic inflammation and inflammation-driven tumorigenesis. These findings highlight the importance of this pathway in restraining excessive inflammatory responses (204).

At the enzymatic level, several additional targets have emerged. Inhibition of acyl-CoA dehydrogenase short-chain (ACADS) promotes M2 macrophage polarization. ACADS deficiency drives TAMs toward an M2 phenotype, suggesting that targeting this enzyme may help resolve persistent inflammation (205). In addition, methionine sulfoxide reductase B1 (MsrB1) regulates redox homeostasis in pro-inflammatory macrophages. MsrB1 deficiency alters glucose and pyruvate utilization, creating a distinct metabolic signature associated with hyper-inflammation. Loss of MsrB1 leads to sustained oxidation of glyceraldehyde 3-phosphate dehydrogenase (GAPDH) residue M44, triggering GAPDH aggregation, inflammasome activation, and increased IL-1β secretion. Consequently, MsrB1-knockout mice exhibit heightened susceptibility to LPS-induced sepsis, highlighting the MsrB1-GAPDH axis as a key link between protein redox homeostasis and metabolic control (206).

## 5 Conclusion and perspective

Macrophage polarization is intrinsically linked to metabolic reprogramming. Pro-inflammatory macrophages rely on enhanced glycolysis, which sustains the production of inflammatory mediators such as TNF-α, IL-1β, and ROS. In contrast, anti-inflammatory macrophages predominantly utilize OXPHOS and fatty acid oxidation to support their reparative and immunoregulatory functions. These distinct metabolic programs not only meet the specific energetic demands of each phenotype but also actively shape macrophage effector responses, thereby influencing the trajectory and resolution of inflammatory processes during S-ALI (207, 208). As sepsis progresses, the metabolic landscape of macrophages undergoes dynamic temporal shifts: the early hyperinflammatory phase is characterized by enhanced glycolysis, while the late immunosuppressive phase exhibits impaired OXPHOS and FAO, with profound defects in the carnitine shuttle observed specifically in non-survivors (62, 160). This biphasic metabolic trajectory underscores the necessity of considering disease stage when designing therapeutic interventions.

The traditional M1/M2 binary classification, while heuristically useful, warrants critical reflection. As discussed above, macrophage activation *in vivo* encompasses a fluid continuum of functional states shaped by the convergence of diverse signals, intracellular pathways, and epigenetic modifications (34–36). The binary model, despite its experimental convenience, fails to capture the intermediate and hybrid phenotypes that are particularly prevalent in complex inflammatory environments such as the septic lung. A related and equally important consideration is the heterogeneity of macrophage populations within the lung (209). Alveolar macrophages, interstitial macrophages, and recruited monocyte-derived macrophages possess distinct developmental origins, transcriptional programs, and homeostatic functions. However, the extent to which these subpopulations undergo divergent metabolic reprogramming during S-ALI remains poorly characterized. In the current literature, most mechanistic insights have been derived from reductionist models employing BMDMs, peritoneal macrophages, or immortalized cell lines, and it remains unclear how faithfully these systems recapitulate the metabolic behavior of specific lung-resident subsets. Moreover, local microenvironmental factors, including oxygen tension, pH, nutrient availability, and extracellular matrix composition, are likely to exert profound influences on macrophage metabolism within different compartments of the injured lung, yet these variables are rarely considered in experimental designs. Future studies should therefore adopt multidimensional analytical approaches, including single-cell transcriptomics, metabolomics, and spatial profiling, to more precisely define the macrophage activation landscape in S-ALI, to identify functionally discrete subsets that may serve as specific therapeutic targets, and to incorporate physiologically relevant microenvironmental conditions into experimental systems.

Beyond the complexity of macrophage heterogeneity, it is increasingly clear that the metabolic pathways governing macrophage function in S-ALI do not act in isolation but rather form a highly interconnected network. As highlighted throughout this review, the TCA cycle serves as a central metabolic hub where these pathways converge (121): citrate derived from glycolysis feeds fatty acid synthesis and itaconate production (124, 129, 130), glutamine sustains the TCA cycle and regulates inflammatory signaling (131, 183), and fatty acid oxidation influences the availability of acetyl-CoA for epigenetic modifications (80, 174). The HIF-1α pathway further exemplifies this interconnectedness, as it is regulated by metabolites derived from glycolysis, the TCA cycle, and fatty acid metabolism (80, 122). Consequently, therapeutic strategies targeting a single metabolic node may induce compensatory shifts in other pathways, potentially limiting their efficacy or producing unintended consequences (178, 179). Future studies should therefore adopt a systems-level approach to dissect the coordinated regulation of metabolic networks in septic macrophages, and clinical translation efforts should consider multi-target interventions that restore overall metabolic homeostasis rather than isolated pathway activity. This integrative perspective will be essential for advancing the next generation of immunometabolic therapies for S-ALI.

In assessing the translational potential of the therapeutic strategies discussed, several limitations must be frankly acknowledged. Natural compounds and traditional Chinese medicine formulations, such as cynaroside (68), rhein (91), eriocitrin (92), Xijiao Dihuang decoction (106), and Taohe Chengqi decoction (107), offer the advantage of pleiotropic action, simultaneously modulating multiple metabolic and inflammatory pathways, and many have a history of clinical use that provides preliminary safety evidence. However, the active components of these formulations are often incompletely characterized, their direct molecular targets remain to be definitively established, and systematic pharmacokinetic, toxicity, and cell-type selectivity data are generally lacking. Conversely, specific metabolic inhibitors such as 2-DG, 3PO, and etomoxir provide well-defined molecular targets and have been instrumental in establishing mechanistic proof-of-concept (61, 93, 178). Yet, their clinical translation is hindered by broad cytotoxicity arising from the ubiquitous importance of metabolic pathways across cell types and a lack of macrophage selectivity. Importantly, even when target engagement is achieved at the cellular level, single-node metabolic interventions may still fail owing to compensatory reprogramming of the broader metabolic network (179). To circumvent these selectivity challenges, current preclinical studies have employed *in vitro* models using defined macrophage populations to demonstrate cell-specific effects, while *in vivo* approaches have largely relied on macrophage-specific genetic manipulations (70, 172, 173, 204). However, these strategies cannot fully recapitulate the complexity of the septic microenvironment, and genetic approaches are not readily translatable to clinical settings. Encouragingly, emerging delivery technologies are beginning to bridge this gap. Aerosolized CRISPR/Cas9 nanotherapeutics enable lung-targeted metabolic gene editing specifically in pulmonary macrophages (94). Biomimetic nanoplatforms exploit alveolar macrophage membrane tropism for cell-specific drug delivery (119). pH-responsive nanoparticles selectively release inhibitors within the acidic inflammatory microenvironment characteristic of septic lungs (118). M2 macrophage-derived exosomes achieve sustained and macrophage-selective pulmonary delivery of metabolic regulators through homotypic recognition (117). These innovations represent important progress toward achieving macrophage-selective metabolic modulation while minimizing systemic off-target effects, and their continued development will be essential for translating immunometabolic therapies from preclinical models to clinical practice.

As the field moves toward clinical translation, several additional considerations warrant attention. Given the fundamental species-specific differences in metabolic regulation, such as the dispensability of FAO for human M2 polarization, in contrast to its critical role in mice (178), findings obtained from rodent systems should be extrapolated to human S-ALI with appropriate caution. Future preclinical studies should therefore prioritize the use of human primary cells, clinical samples, and humanized models to validate metabolic targets identified in mouse studies. Furthermore, the timing of therapeutic intervention must be carefully calibrated to the evolving metabolic landscape of sepsis, as a strategy that is protective during the early hyperinflammatory phase may be detrimental during the late immunosuppressive phase. Finally, because metabolic intermediates often display disease-associated alterations, their detection holds considerable promise for diagnostic applications (210). Although advances in metabolic profiling technologies are rapidly expanding current knowledge, substantial gaps remain, particularly regarding the epigenetic regulation of immune cell metabolism, metabolite-driven post-translational modifications, and the intricacies of metabolic signaling networks. Given its central role in shaping immune responses, macrophage immunometabolic reprogramming represents a promising therapeutic avenue for immune-mediated disorders (211). Addressing these gaps through rigorous, multidisciplinary investigation may ultimately yield innovative strategies for the treatment of sepsis-associated acute lung injury.
